# Can oxygen anosmia extend lifespan?

**DOI:** 10.18632/aging.101337

**Published:** 2017-11-30

**Authors:** Rachel Abergel, Einav Gross

**Affiliations:** Department of Biochemistry and Molecular Biology, IMRIC, Faculty of Medicine, The Hebrew University of Jerusalem, Jerusalem, 9112102 Israel

**Keywords:** C. elegans, lifespan, soluble guanylate cyclase, NPR-1, oxygen sensing, reactive oxygen species, hypoxia

All aerobic animals use oxygen (O_2_) to generate energy through mitochondrial respiration. Moreover, the metabolism of O_2_ in cells creates reactive oxygen species (ROS) that are important for regulating diverse and important physiological processes such as autophagy and wound healing [1]. However, excessive production of ROS can damage various biological molecules causing tissue destruction and cell death. Therefore, tight regulation of O_2_ homeostasis is crucial for the healthspan and lifespan of all aerobic animals.

To maintain O_2_-homeostasis, aerobic animals developed mechanisms for O_2_-sensing. The O_2_-sensors regulating these mechanisms are fine-tuned to respond to changes in O_2_ level and so act as safeguards protecting cells both from life-threatening shortage of ATP and from oxidative stress. However, despite this important role, our knowledge about O_2_-sensor function in healthspan and lifespan regulation is limited.

The atypical soluble guanylate cyclases (sGCs) GCY-35 and GCY-36 are O_2_-sensors that facilitate the escape of the nematode *Caenorhabditis elegans* (*C. elegans*) from hyperoxia (O_2_>12%) [2]. These O_2_-sensors create a functional heterodimer in the O_2_-sensing neurons AQR, PQR, and URX. Upon O_2_-binding, the activity of GCY-35/GCY-36 is increased and thus more cyclic GMP (cGMP) is generated. cGMP triggers the opening of the TAX-2 and TAX-4 cyclic nucleotide-gated channels, which facilitates calcium entry and thus the activation of AQR, PQR, and URX [2]. The hyperoxia avoidance response is inhibited by the function of the neuropeptide receptor NPR-1 in the presence of bacteria (the *C. elegans* food source). Therefore, animals bearing the dominant allele of *npr-1*, *npr-1(215V)*, move slowly on food and do not accumulate on the bacterial lawn border. However, animals bearing either the recessive *npr-1(215F)* allele or the loss-of-function allele *npr-1(ad609),* hereafter referred to as *npr-1(−)*, move quickly on food and aggregate together on the bacterial lawn border (aggregation behaviour). The consumption of O_2_ in the aggregate creates O_2_ conditions preferred by *C. elegans*, with 6.4% O_2_ in the middle of the clump [3]. Such conditions are less oxidizing and therefore may provide a healthier living environment for the worm. With this thought in mind, we set out to explore the function of NPR-1 and GCY-35/GCY-36 in health-span and lifespan.

The lifespan of *npr-1(−)* worms was slightly, but significantly, longer than that of N2 animals bearing the *npr-1(215V)* allele [4]. This result fitted our prediction that the aggregation behaviour can create a “healthier O_2_ microenvironment” and thus increases the lifespan of *npr-1(−)* worms. However, the next step in our studies exemplified Huxley's saying: “The great tragedy of Science - the slaying of a beautiful hypothesis by an ugly fact”. We predicted that the *gcy-35(ok769)* deletion allele (which suppresses the aggregation behaviour), hereafter referred to as *gcy-35(−)*, would decrease the lifespan of *npr-1(−)* animals back to N2 worms' lifespan. However, the result was the opposite. The lifespan of *gcy-35(−);npr-1(−)* worms was profoundly longer than that of both N2 and *npr-1(−)* worms [4]. In fact, the joint effect of *gcy-35(−)* and *npr-1(−)* on worm lifespan is synergistic, since *gcy-35(−)* worm lifespan was similar to N2 and *npr-1(−)* by itself elicits only a small increase in lifespan.

Why does joint inhibition of *gcy-35* and *npr-1* increase lifespan? Previous studies show that low O_2_-levels increase the lifespan of worms and flies. For example, 1% O_2_ extends the lifespan of *C. elegans* and moderate hypoxia (10% O_2_) extends the maximum lifespan of *Drosophila melanogaster* [5]. Since *gcy-35* is essential for hyperoxia-sensing in *npr-1(−)* worms, we hypothesize that *gcy-35(−);npr-1(−)* worms “feel” as if they are in hypoxia. As a result, adaptation mechanisms for hypoxia are activated, including defences against bacterial toxicity and DNA damage. These mechanisms increase the healthspan and lifespan of *gcy-35(−);npr-1(−)* worms at 21% O_2_ (Figure 1). Our data support this hypothesis, Firstly, the hypoxia inducible transcription factor HIF-1 is required for the lifespan lengthening of *gcy-35(−);npr-1(−)* worms. HIF-1 is a key regulator of adaptation to hypoxia and is important for innate immunity [6]. Secondly, we show that the sGCs *gcy-31* and *gcy-33* are required for lifespan extension. These sGCs act reciprocally to GCY-35/GCY-36 and are activated at low O_2_-levels [4]. Genetic ablation of the BAG neurons in which they are expressed inhibits the lifespan extension of *gcy-35(−);npr-1(−)* worms, further supporting the hypothesis that hypoxia sensing is im-portant for the lifespan lengthening. Finally, *gcy-35(−);npr-1(−)* worms are more resistant to both *Pseudomonas aeruginosa* (PA14) bacteria and ultraviolet (UV) irradiation compared to N2 and *npr-1(−)* controls, suggesting that defence mechanisms against pathogenic bacteria and DNA damage are activated in *gcy-35(−);npr-1(−)* worms. Indeed, innate immunity genes are upregulated in *gcy-35(−);npr-1(−)* worms compared to N2 and *npr-1(−)* animals [4].

**Figure 1:**
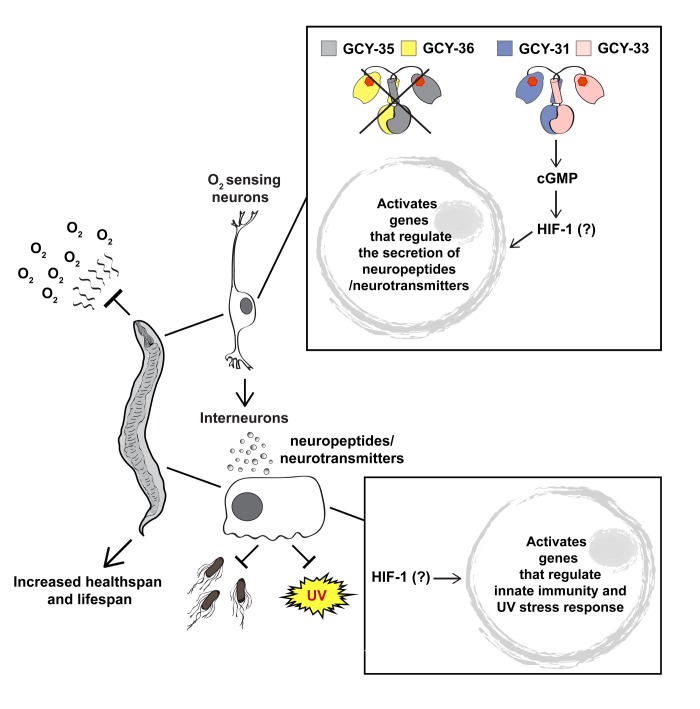
A schematic illustration presenting a model for the increased lifespan and healthspan of *gcy-35(−);npr-1(−)* animals. In the absence of *gcy-35*, the O_2_-sensing neurons AQR, PQR, and URX are not activated by O_2_. Our data suggest that GCY-31 and GCY-33 activate a HIF-1-dependent signalling pathway in which neuropeptide and neurotransmitter induce defence responses against bacteria and UV damage in remote tissues. Therefore, although the worm is in a hyperoxic environment, it can benefit from defences activated by hypoxia signalling. The question mark represents our uncertainty about the tissues/cells in which HIF-1 activity is required.

In conclusion, our data suggest that worms can enjoy the beneficial effect of hypoxia signaling without actually being in hypoxia. By genetically manipulating the worm not to smell high O_2_, we can activate defence mechanisms that extend both healthspan and lifespan. Intriguingly, previous studies show that the smell of food can decrease the lifespan of worms and flies (when fed on a calorie-restricted diet) and a recent paper described how ablation of olfactory sensory neurons in mice makes them resistant to obesity caused by an enriched-fat diet [7]. Temporary exposure to high altitude results in lose-of-appetite and consequently weight loss in people (altitude anorexia). Although the molecular mechanism underlying altitude anorexia is not well understood, it appears that hypoxia is the causative agent that controls the change in appetite. A fascinating direction for future studies will be to determine the interplay between oxygen sensing, appetite regulation and longevity.

## References

[1] Finkel T (2011). J Cell Biol.

[2] Gray JM (2004). Nature.

[3] Rogers C (2006). Curr Biol.

[4] Abergel R (2017). Aging Cell.

[5] Rascón B, Harrison JF (2010). J Exp Biol.

[6] Zuckerman B (2017). Free Radic Biol Med.

[7] Garrison JL, Knight ZA (2017). Science.

